# Editorial: Highlights in digital mental health 2021/22

**DOI:** 10.3389/fdgth.2022.1093375

**Published:** 2023-01-18

**Authors:** Anabel De la Rosa-Gómez, Karin Waldherr

**Affiliations:** ^1^ Faculty of Higher Studies Iztacala, National Autonomous University of Mexico, Tlalnepantla, Mexico; ^2^ Ferdinand Porsche FernFH, Distance Learning University of Applied Sciences, Wiener Neustadt, Austria

**Keywords:** COVID-19, telemental health (TMH), chatbot, meta-analysis, engagement, depression, older adults, social isolation

**Editorial on the Research Topic** Highlights in digital mental health 2021/22

The section *Digital Mental Health* publishes research focused on prevention and treatment of mental disorders that is at the intersection of psychiatry, psychology, and technology. Due to it's multidisciplinary character, the section appears in *Frontiers in Digital Health* as well as in *Frontiers in Psychiatry*.

In recent years, there has been an increase in initiatives focused on the promotion and intervention mediated by technologies and that have favored access and dissemination of effective interventions regardless of distances, physical and social barriers and with this, reaching people who otherwise would not receive attention. The COVID-19 pandemic has led to a sudden shift in health and care services, moving from face-to-face to online, challenging mental health professionals to quickly adapt to meet user needs. The pandemic presented a double challenge because not only was it necessary to design and develop interventions to meet the demand for mental health services, but these interventions had to be adapted to the requirements of a population that was in confinement. Therefore, the development of projects that provided remote care to the general population was extremely relevant. However, given the emerging transition, it is relevant to point out some ethical and privacy considerations related to the use of technologies applied to psychological interventions aimed at confidentiality, competence and responsibility in their implementation.

For this purpose, this collection includes a selection of articles, which gained high interest in 2021/2022 (see Table 1).

**Table 1:** Number of views and citations by 04 Jan 2023 of the articles included in the collection (authors appear in the order of publication date).

Author (s)	Publication date	Views	Citations
Lina Gega & Elias Aboujaoude	14 Sep 2021	7,104	1
Gilly Dosovitsky & Eduardo Bunge	06 Oct 2021	2,930	2
Daniel Gan, Lauren McGillivray, Jin Han, Helen Christensen & Michelle Torok	04 Nov 2021	5,244	5
Heather Belanger & Mirène Winsberg	22 Aug 2022	1,198	–

The topics that attracted interest include an opinion article discussing digital technologies as mediator of effects of the COVID-19 pandemic on mental health, a meta-analysis and original research to explore factors that might have an influence on the outcomes of digital mental health interventions as well as user experiences with a chatbot.

The importance of digital technologies increased sharply during the pandemic. In their opinion article, Gega & Aboujaoude therefore discuss how digital technologies have mediated negative, positive and neutral effects of the COVID-19 pandemic on mental health. Acceptance of digital treatment options for mental illnesses increased during the pandemic and the possibility to stay socially connected during lockdowns by means of digital technologies helped to neutralize some negative effects on mental health. However, the pandemic had pronounced negative effects for certain population groups, and limited access to digital technologies for these groups may have further exacerbated health disparities. The authors thus conclude that we should harness the benefits of digital technologies for mental health while reducing associated risks and inequities to reach those most in need for mental health care.

The three original research papers add to the issues raised in the opinion article by Gega & Aboujaoude.

During the pandemic older adults were one group with high need for mental health care. They were among the most vulnerable population groups and feared infection through personal contacts, which contributed to social isolation and increased depressiveness. The article by Belanger and Winsberg deals with the question whether treatment effects of psychiatric care for depression *via* a telemental health platform for older adults are comparable to those for younger adults. The results are promising, showing that older adults who chose to receive treatment through a telemental health platform showed a similar improvement in depression symptoms as younger adults. This is therefore an example of how digital technologies can help to mitigate the negative impact of a pandemic for a vulnerable group.

In order to achieve intervention outcomes in digital health, engagement has been recognized as important (1, 2). However, there was only a narrative synthesis on the relationship between engagement and intervention outcomes available (3), but no meta-analysis on the effects of higher engagement on mental health outcomes in digital mental health interventions. Gan et al. closed this research gap. Their already urgently needed work underpins the relationship between engagement and intervention outcomes and the necessity of conducting more research focusing on engagement and targeted strategies to increase user engagement.

Users' experiences with a chatbot to combat loneliness and social isolation for adults and older adults were examined by Gilly Dosovitsky & Eduardo Bunge. The results regarding satisfaction are in line with previous findings: most users who answered the question were satisfied with the chatbot. Qualitative analyses revealed that some users prefer human interactions. Others were dissatisfied, because the chatbot misunderstood the user and gave irrelevant responses. It was found that many users tended to personify the chatbot and assigned human traits to it. Furthermore, users were able to bond with the chatbot. Although not everyone is approachable with chatbots they are promising approaches to support people who are amenable to them regarding mental health issues. However, as (4) have pointed out, prerequisite for chatbots to be used in mental health care is their implementation in compliance with quality and ethical standards as well as a better evidence base (4).

Thus, the evidence seems promising, remote interventions with technological implementations have turned out to be novel and innovative; however, not all countries have achieved robust development and research in the field. The articles in this collection identified several promising areas for future research including development of targeted strategies to increase reach of population groups most in need for digital mental health interventions and to increase user engagement as well as further research on the use of chatbots in mental health care. It is necessary to generate a greater degree of research and technological development because it is incipient, not only to cover the existing demand for care after the COVID-19 pandemic, but also to reduce the risk factors that increase the possibility of developing mental health problems or exacerbate pre-existing ones.
